# Myocardial Lipid Metabolism Imbalance: The Pathological Core and Novel Diagnostic‐Therapeutic Directions of Cardiovascular Diseases

**DOI:** 10.1002/jbt.71033

**Published:** 2026-07-27

**Authors:** Peiyun Xie, Qun Zeng, Hongrui Wang, Meihua She

**Affiliations:** ^1^ Department of Biochemistry and Molecular Biology, Hengyang Medical School University of South China Hengyang Hunan China

**Keywords:** cardiac lipid metabolism, cardiovascular diseases, fatty acid oxidation, lipotoxicity, targeted therapy

## Abstract

Cardiac lipid metabolism is fundamental to myocardial energy homeostasis, with fatty acid oxidation (FAO) supplying the majority of ATP in the healthy adult heart. This review synthesizes the core regulatory network governing cardiac lipid metabolism, encompassing lipid droplet dynamics mediated by perilipins (e.g., Plin5, Plin2), fatty acid uptake via CD36, systemic lipid modulation by apolipoproteins (e.g., APOC3), and the central energy‐sensing AMPK/PGC‐1α/PPARα axis. Dysregulation of this network initiates a self‐perpetuating lipotoxic cycle, characterized by the accumulation of toxic lipid intermediates (e.g., diacylglycerols, ceramides), oxidative stress, and inflammatory activation, which serves as a common pathological mechanism across diverse cardiovascular diseases (CVDs), including atherosclerosis, heart failure, diabetic cardiomyopathy, and ischemic injury. Emerging from this mechanistic understanding is a promising landscape of biomarkers—such as specific ceramide species, the ApoB/ApoA‐1 ratio, and circulating perilipins—and targeted therapeutic strategies, including APOC3 inhibitors, SGLT2 inhibitors, and Plin5‐directed therapies. Future advances will depend on integrating multi‐omics technologies and precision medicine approaches to tailor interventions to specific metabolic phenotypes, thereby opening new avenues for the prevention and treatment of CVDs.

## Graphical abstract

In cardiac cells, Plin5/AMPK regulate lipid homeostasis; excess CD36‐mediated uptake drives lipotoxicity, mitochondrial dysfunction, and CVDs (e.g., heart failure). Biomarkers (ApoB/ApoA‐1) and therapies (SGLT2 inhibitors) target this cascade.

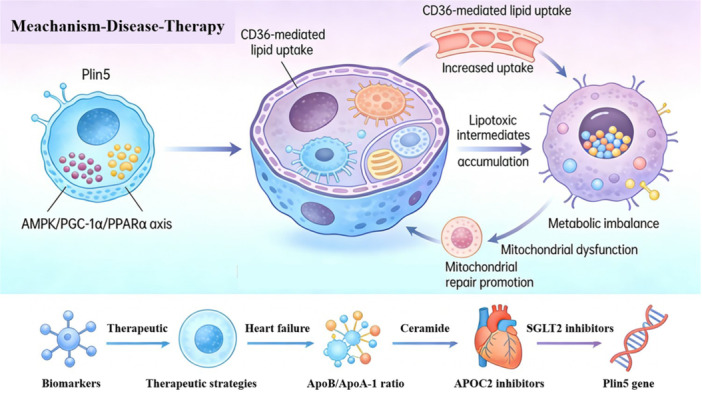

## 1 Introduction

The heart ranks among the most energy‐demanding organs, with its continuous pumping function critically dependent on a stable and highly efficient supply of ATP [1, 2, 3]. In the adult mammalian heart, FAO stands as the predominant source, accounting for 60%–70% of ATP production under physiological conditions [3, 4, 5]. The maintenance of cardiac lipid metabolic homeostasis—encompassing fatty acid uptake, esterification into lipid droplets (LDs), mobilization via hydrolysis, and final mitochondrial oxidation—is decisive for preserving myocardial structure and function [6, 7]. Maintaining cardiac lipid homeostasis requires both exogenous lipid utilization and endogenous de novo lipogenesis (DNL). While DNL provides essential structural and storage lipids under physiological conditions, its pathological overactivation promotes lipotoxicity, driving cardiovascular diseases including atherosclerosis and myocardial hypertrophy [8].

**FIGURE 1:**
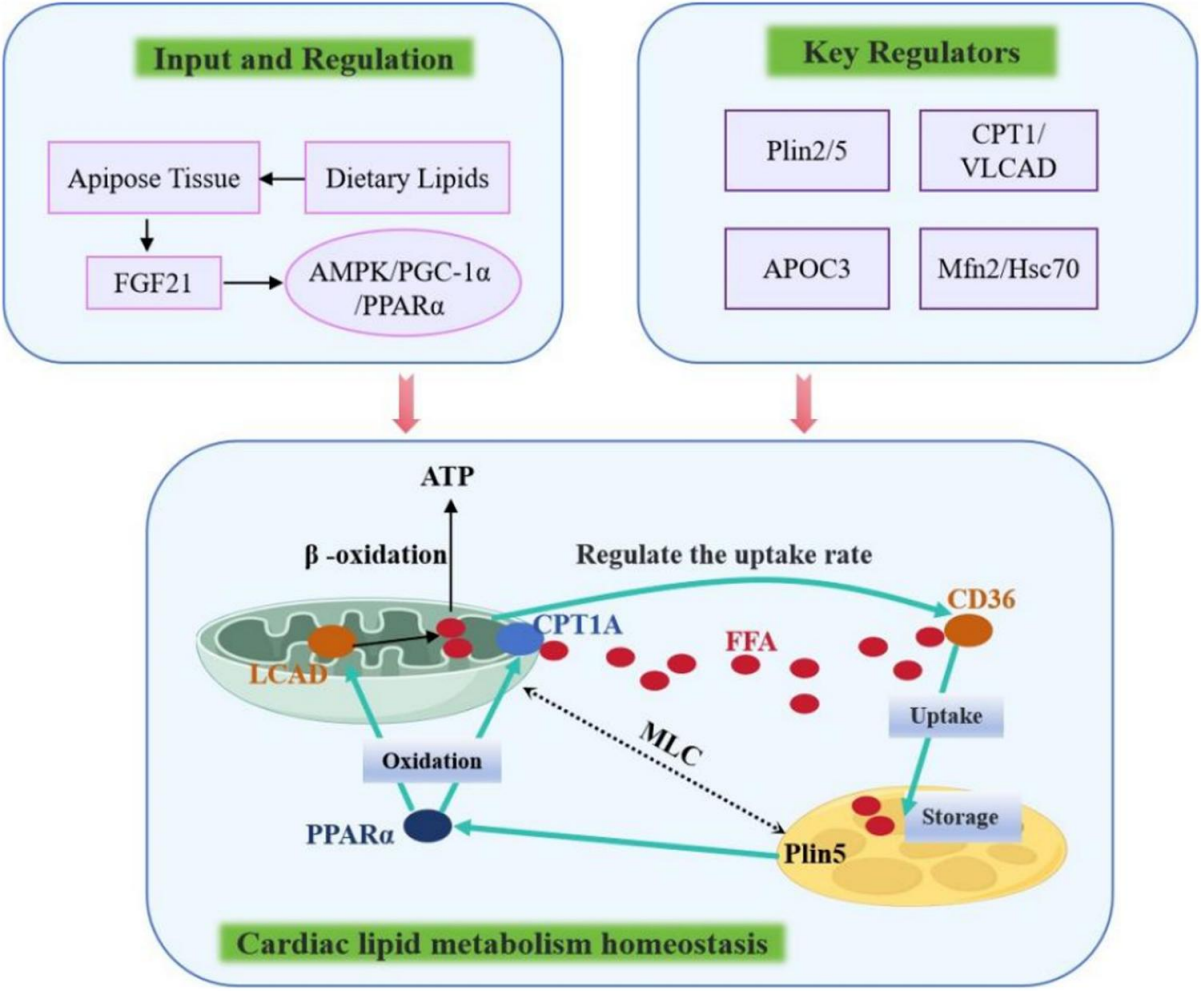
Regulatory architecture of cardiac lipid metabolism in the healthy heart. This schematic integrates the key processes and regulators of free fatty acid (FFA) handling in cardiomyocytes under physiological conditions. Circulating FFAs, modulated by apolipoproteins (e.g., APOC3), are primarily taken up via the transporter CD36. Intracellular FFAs are either esterified and stored in LDs coated with Plin5 or directed to mitochondria for β‐oxidation and ATP production. The physical Mitochondria‐LDs contact site (MLC), facilitated by Plin5 and Mfn2, enables efficient channeling of FFAs. Systemic hormones (e.g., FGF21) and the core energy‐sensing AMPK/PGC‐1α/PPARα axis centrally coordinate this network to match energy supply with demand.

This homeostasis is dynamically regulated by a suite of key molecules, including LDs‐coating proteins such as Plin5 [6, 9], the fatty acid transporter CD36 [10, 11], apolipoproteins including APOC3 and Apolipoprotein B (ApoB) [12, 13], the metabolic hormone fibroblast growth factor 21 (FGF21) [14], and the core energy‐sensing AMPK/PGC‐1α/PPARα axis [15, 16]. These components form a coordinated regulatory system that precisely directs the balance between lipid storage and oxidation (Figure 1).

Disruption of this equilibrium leads to abnormal intracellular lipid accumulation, triggering lipotoxicity—characterized by excessive buildup of toxic lipid intermediates like diacylglycerols (DAG) and ceramides [16, 17, 18]. This triggers a pathological cascade involving oxidative stress, insulin resistance, inflammatory responses, and apoptosis, final leading to myocardial dysfunction and structural remodeling [9, 12, 19]. Substantial evidence confirms that dysregulated cardiac lipid metabolism is a common pathological thread linking CVDs, including atherosclerosis [20, 21], heart failure (particularly heart failure with preserved ejection fraction, HFpEF) [19, 22] to DCM [16, 23], myocardial ischemia/reperfusion (I/R) injury [9, 24], and drug‐induced cardiotoxicity [25, 26].

Recent advances in multi‐omics technologies and molecular imaging have profoundly expanded our understanding of this regulatory network. Newly identified regulatory molecules, such as non‐coding RNAs (ncRNAs) [27] and organelle interaction proteins [7], continue to emerge. Concurrently, promising clinical biomarkers (ApoB/ApoA‐1 ratio [28] and specific ceramide species [29]) and targeted therapeutic strategies (APOC3 inhibitors [12], SGLT2 inhibitors [16, 30], and Plin5‐based gene therapies [2, 25]) are advancing toward clinical exploration.

Translational Considerations and Species‐Specific Limitations. While mechanistic insights from animal models have been invaluable, important metabolic differences exist between commonly used rodent models and humans that may limit predictive value for clinical outcomes. Mice and rats exhibit higher baseline FAO rates, distinct lipid profiles (e.g., higher HDL‐to‐LDL ratios), and different responses to metabolic stress compared to humans [31, 32]. Additionally, the lack of certain genetic risk factors in standard strains and differences in cardiac regenerative capacity complicate direct translation. Therefore, findings from preclinical studies require cautious interpretation and validation in human‐relevant systems, including induced pluripotent stem cell‐derived cardiomyocytes and large animal models [31].

Recent advances in multi‐omics technologies, molecular imaging, and data‐driven modeling have profoundly expanded our understanding of this regulatory network. Newly identified regulatory molecules, such as non‐coding RNAs (ncRNAs) [27] and organelle interaction proteins [7], continue to emerge. Concurrently, promising clinical biomarkers (ApoB/ApoA‐1 ratio [28] and specific ceramide species [29]) and targeted therapeutic strategies (APOC3 inhibitors [12], SGLT2 inhibitors [16, 30], and Plin5‐based gene therapies [2, 25]) are advancing toward clinical exploration.

This review aims to systematically synthesize the core regulatory elements of cardiac lipid metabolism, conduct a mechanistic exploration of their pivotal roles in the pathogenesis of CVD, and present a forward ‐ looking perspective on the clinical translation of related biomarkers and targeted therapies.

## 2 Core Regulatory Network of Cardiac Lipid Metabolism

### 2.1 Intracellular Lipid Storage and Mobilization: LDs and Perilipins

LDs are now recognized as highly dynamic organelles that function as crucial metabolic hubs in cardiomyocytes, involved in lipid buffering, energy supply, and signal transduction [6]. Their functional regulation is centrally mediated by the perilipin protein family coating their surfaces, with Plin2 and Plin5 exhibiting distinct and critical roles in the heart.

#### 2.1.1 Plin5: The Oxidation Promoter

Plin5 is highly expressed in oxidative tissues like the heart, where it acts as a key guardian of myocardial lipid homeostasis [2, 6, 9]. Through its C‐terminal domain, Plin5 anchors to mitochondria, facilitating the formation of LDs‐mitochondria contact sites. This physical connection creates an efficient channel for directed fatty acid transport from storage pools to oxidation sites, thereby enhancing transfer efficiency and preventing cytosolic lipid accumulation and associated lipotoxicity [2, 7]. Under basal conditions, Plin5 coats LDs and potently suppresses lipolysis—reducing its rate by up to 65%—by competitively binding to adipose triglyceride lipase (ATGL) and its co‐activator, CGI‐58. During high‐energy‐demand states, PKA‐mediated phosphorylation releases this inhibition, initiating lipolysis to meet urgent energy needs [2, 33]. This regulatory mechanism underpins the protective role of Plin5 in models of myocardial I/R injury, where its overexpression can reduce infarct size by up to 58% [2, 9].

#### 2.1.2 Plin2: The Storage Promoter

In contrast to Plin5, Plin2 primarily promotes lipid accumulation. In macrophages, elevated Plin2 expression accelerates intracellular cholesteryl ester accumulation, driving foam cell formation and atherosclerosis progression by inhibiting neutral cholesteryl ester hydrolase (nCEH) activity and ABCA1‐mediated cholesterol efflux [6, 20, 34]. In cardiomyocytes, Plin2 also facilitates lipid storage; its specific knockout can suppress AMPK signaling and lipophagy, leading to triglyceride and DAG accumulation and worsened post‐infarction cardiac function, suggesting a role in maintaining energy homeostasis under stress [35].

#### 2.1.3 The Lipolytic Machinery and Triglyceride Synthesis

The lipolytic machinery itself is vital. ATGL serves as the rate‐limiting enzyme for lipolysis, with its activity precisely regulated by CGI‐58. Dysfunction of either protein is linked to severe cardiomyopathy, underscoring the importance of the dynamic balance at the LDs surface [17].

Beyond its role in storage, LDs and their associated proteins are increasingly regarded as integrated signaling platforms. However, their interactions with other organelles, such as the endoplasmic reticulum (ER) and lysosomes, in the heart remain an underexplored frontier. The synthesis of triglycerides (TG), the core component of LDs, is also precisely regulated. Studies have shown that the key enzymes in the final step of TG synthesis ‐ diacylglycerol acyltransferase 1/2 (DGAT1/2) ‐ are partially redundant in function in the heart. Inhibiting any subtype alone has a mild impact on the heart, but combined inhibition can completely block the turnover of TG and effectively reduce lipid accumulation in the heart in the context of a high‐fat diet. This provides experimental evidence for regarding DGAT as a potential therapeutic target for high‐fat‐related heart diseases [36].

### 2.2 Context‐Dependent Regulation and Plasticity of Perilipins

#### 2.2.1 Functional Plasticity of Plin5

Intriguingly, in specific contexts like alcoholic cardiomyopathy, Plin5 downregulation and reduced LDs‐mitochondria contacts appear adaptive, serving to avoid oxidative damage from excessive fatty acid flux, illustrating its functional plasticity [37]. The functional background dependence of Plin5 is equally significant during the aging process. Studies have shown that aging itself leads to the down‐regulation of Plin5 expression in mouse cardiomyocytes, and in the basal state, it relies on lysosomal lipolysis to maintain balance. A high‐fat diet can induce a compensatory increase in Plin5 in elderly cardiomyocytes, but this increase instead exacerbates lipid accumulation, which is in stark contrast to the flexibility response maintained by activating lipid metabolism enzymes in young mice. This reveals that age is a key factor in determining the functional output of Plin5 and its metabolic consequences [38]. This context‐dependent role of Plin5 suggests that promoting LDs‐mitochondria coupling is not universally beneficial, and future efforts should define the precise metabolic conditions that dictate its optimal functional output.

#### 2.2.2 Fine‐Tuning of Plin5 by Endogenous Signals

Notably, the function of Plin5 can be precisely regulated by endogenous signaling molecules. For instance, acetylcholine has been shown to exert dual protective effects in palmitate‐induced lipotoxicity models by upregulating Plin5 expression: on one hand, it activates LDs lipolysis to reduce toxic lipid accumulation; on the other hand, it significantly enhances LDs‐mitochondria contact, optimizes fatty acid oxidation and improves mitochondrial function, ultimately inhibiting cardiomyocyte apoptosis. This provides a novel strategic perspective for targeting the “cholinergic signaling‐Plin5‐LDs metabolism” axis in the treatment of lipotoxic cardiomyopathy [39].

#### 2.2.3 Non‐Canonical Pathways of Lipolysis Regulation

Notably, non‐canonical pathways also regulate lipid mobilization, as demonstrated by okadaic acid‐induced perilipin phosphorylation and lipolysis activation through PP1/PP2A inhibition [40].

### 2.3 Fatty Acid Uptake and Intracellular Trafficking

#### 2.3.1 CD36: The Principal Transporter and Genetic Variability

The fatty acid translocase CD36 is the principal mediator of long‐chain fatty acid uptake in cardiomyocytes, facilitating approximately 70% of total uptake and serving as the essential gatekeeper for myocardial lipid supply [10, 11]. CD36 activity is regulated at multiple levels: acute stimuli (e.g., insulin, AMPK activation) prompt its translocation to the plasma membrane, while long‐term expression is modulated by transcription factors like PPARα.

Genetic Variability and Disease Susceptibility. Beyond regulatory mechanisms, genetic variants significantly influence CD36 function and disease susceptibility. Single nucleotide polymorphisms (SNPs) in the CD36 gene (e.g., rs1527483, rs3211938) alter baseline expression levels and fatty acid uptake capacity among individuals, potentially modifying susceptibility to cardiometabolic diseases [10, 41]. These variants can lead to either insufficient activity (impairing physiological fatty acid utilization) or excessive activity (promoting lipotoxicity under metabolic stress). This genetic heterogeneity complicates therapeutic targeting and may explain variable responses to metabolic interventions across patient populations [10, 41].

Under pathological conditions such as obesity and diabetes, persistent lipid overload causes abnormal CD36 retention at the sarcolemma. This leads to chronically elevated fatty acid uptake that surpasses oxidative capacity, resulting in toxic lipid intermediate accumulation, insulin resistance, and myocardial dysfunction—core features of lipotoxicity [11, 16, 42]. However, the therapeutic targeting of CD36 is complicated by its pleiotropic roles in immunity and angiogenesis, necessitating the development of cardiac‐specific modulation strategies to avoid off‐target effects [41, 43].

#### 2.3.2 FATP2 and Other Uptake Routes

Beyond CD36, fatty acid transport protein 2 (FATP2) also contributes significantly to myocardial fatty acid uptake. Its expression is markedly upregulated in obesity, working in concert with CD36 to mediate excessive uptake and exacerbate lipotoxic injury [44]. Notably, the GLP‐1 receptor agonist semaglutide suppresses myocardial Slc27a2 expression, providing a mechanistic rationale for therapeutic targeting [44].

#### 2.3.3 Intracellular Trafficking and Organelle Contacts

Following cytosolic entry, efficient fatty acid trafficking to mitochondria is crucial. Proteins like mitofusin‐2 (Mfn2) and Plin5 facilitate the formation of mitochondria‐LDs membrane contact sites, which act as molecular bridges for direct fatty acid transfer, preventing diffuse cytosolic accumulation and counteracting lipotoxicity [2, 7, 17].

#### 2.3.4 Systemic Neurohormonal Regulation

Abnormal fatty acid metabolism not only stems from the imbalance of transporters such as CD36 themselves, but is also strongly influenced by systemic signals (such as stress). For instance, in unpredictable stress models, the activation of cholecystokinin receptors (CCK1R/CCK2R) can exacerbate the disorder of myocardial fatty acid metabolism, while its knockout can improve stress‐induced myocardial injury by correcting the abnormalities of metabolites such as arachidonic acid and cholesterol, revealing the remote regulation of cardiac lipid metabolism by the neurohormonal system [45].

### 2.4 Mitochondrial Fatty Acid Oxidation and Energy Sensing

#### 2.4.1 Key Enzymes of β‐Oxidation

Following their cytosolic entry, fatty acids undergo mitochondrial β‐oxidation—the central process governing cardiac energy production. Carnitine palmitoyltransferase I (CPT1), situated on the outer mitochondrial membrane, acts as the rate‐limiting enzyme directly controlling fatty acid flux into mitochondria [5, 16]. The subsequent oxidation process is initiated by very long‐chain acyl‐CoA dehydrogenase (VLCAD), while acetyl‐CoA acyltransferase 2 (ACAA2) operates at the terminal stage. The coordinated expression and activity of these enzymes are crucial for sustaining efficient FAO. Their significant downregulation in pathologies like chronic atrial fibrillation and HF leads to impaired FAO and myocardial energy crisis [46, 47]. Notably, in renal impairment‐associated cardiac dysfunction, uremic toxins can upregulate forkhead box O4 (FOXO4) to suppress ACAA2 expression, thereby inducing myocardial FAO disorders and exacerbating lipid peroxidation [48].

#### 2.4.2 Nuclear Architecture and Metabolic Gene Expression

Recent evidence indicates that nuclear architecture plays an essential role in maintaining metabolic gene expression. Lamin A mutations disrupt the perinuclear heterochromatin compartment, leading to transcriptional repression of key FAO genes including PPARα and CPT1b, thereby compromising myocardial fatty acid oxidation capacity [49].

#### 2.4.3 The Central AMPK/PGC‐1α/PPARα Axis

At the regulatory level, the AMPK/PGC‐1α/PPARα signaling axis constitutes the core regulatory hub. AMPK, the cellular energy sensor, activates under ATP‐depleted conditions to phosphorylate and activate PGC‐1α. This key transcriptional coactivator then synergizes with PPARα to upregulate critical FAO genes (e.g., CPT1, VLCAD), thereby promoting energy production [15, 16, 47]. Suppression of this pathway is a fundamental mechanism in DCM, HF, and atrial fibrillation [16, 47]. Metformin, for instance, demonstrates potential in chronic atrial fibrillation models by activating this axis to ameliorate lipid metabolism and attenuate atrial remodeling [47].

#### 2.4.4 Additional Regulators: SIRT1, FGF21, mTORC2, PGRMC1, and FoxO1

Intertwined with this core axis, sirtuin 1 (SIRT1) deacetylates and activates both PGC‐1α and PPARα, cooperatively enhancing FAO [50]. The systemic metabolic regulator FGF21 exerts cardioprotective effects by activating the myocardial AMPK/PGC‐1α pathway, upregulating key FAO enzymes and promoting utilization of fatty acids and ketone bodies [14]. Furthermore, the mechanistic target of rapamycin complex 2 (mTORC2) promotes mitochondrial fission via a non‐canonical Akt‐independent pathway, recruiting dynamin‐related protein 1 (Drp1), thus providing a novel link between nutrient sensing and mitochondrial dynamics in lipid oxidation regulation [51]. Recent studies have also found that progesterone receptor membrane component 1 (PGRMC1) is a key negative regulatory factor for myocardial energy metabolism. The deficiency of PGRMC1 can maintain ATP levels and alleviate cardiac dysfunction by activating AMPK, which prompts the myocardium to shift from glycolysis to the more efficient oxidation of fatty acids and pyruvate during energy stress [52]. This provides a brand‐new target for metabolic intervention in energy‐deficiency heart failure. Forkhead box transcription factor O1 (FoxO1) is another important metabolic integrator. It dynamically regulates the glycolipid metabolism, oxidative stress, and inflammatory response of endothelial cells and cardiomyocytes via its post‐translational modification state, functioning as a “metabolic switch” in diseases such as atherosclerosis and diabetic cardiomyopathy. The regulation of its activity also serves as a potential therapeutic target [53].

#### 2.4.5 The Dual Role of PPARα Activation

A critical consideration for PPARα ‐ targeted therapy is the timing and context of its activation. Non‐selective agonism in the setting of pre‐existing lipid overload (e.g., early DCM) might paradoxically worsen lipotoxicity by co‐activating fatty acid uptake genes. This complexity is supported by genetic research. In contrast, under metabolic stress conditions (such as a high‐fat diet or fasting), specific knockdown of PPARα expression in the myocardium can instead reduce the abnormal accumulation of myocardial TG by down‐regulating the LDs protein Plin2, without damaging the basal cardiac function. This indicates that in certain lipid overload states, moderately inhibiting (rather than activating) myocardial PPARα may be a beneficial adaptive strategy [54].

#### 2.4.6 Metformin's Multifaceted Actions

Furthermore, recent studies show that metformin significantly mitigates high‐fat diet‐induced myocardial hypertrophy by inhibiting the HIF‐1α/PPAR‐γ pathway. It reduces the expression of HIF‐1α and PPARγ by 45% and 38%, respectively, and downregulates their downstream targets, VEGFA and GLUT1, ultimately alleviating cardiomyocyte hypertrophy, apoptosis, and left ventricular dysfunction [55].

It is important to note that while activation of energy‐sensing pathways like AMPK/PGC‐1α is beneficial in early disease stages, its efficacy in advanced heart failure may be limited by severe underlying mitochondrial damage, highlighting the need for combinatorial strategies that also address mitochondrial quality.

Collectively, FAO and energy sensing pathways constitute a core network that sensitively responds to cellular energy status and regulates fatty acid oxidation flux. Its dysregulation is a common molecular basis for energy metabolic disturbances in cardiac diseases and a valuable therapeutic target.

### 2.5 Systemic and Vascular Interface Regulation of Lipid Supply

#### 2.5.1 Vascular Lipolysis and Its Inhibition

At the vascular interface, lipoprotein lipase (LPL) hydrolyzes TG from circulating lipoproteins (e.g., chylomicrons, VLDL), releasing FFAs for cardiac use [4]. Conversely, APOC3 inhibits LPL activity, impairing the clearance of triglyceride‐rich lipoproteins and representing a key link between systemic lipid disturbances and adverse cardiac metabolic effects [12].

#### 2.5.2 Endothelial Cell Lipid Transport

The lipid supply of the heart is highly dependent on the transport function of capillary endothelial cells. Endothelial cells precisely regulate the delivery of fatty acids and lipoprotein lipids to myocardial tissue through receptors such as CD36, GPIHBP1 and SR‐B1 on their surface. This process undergoes dynamic changes under physiological and pathological conditions, and its functional abnormalities are the key links leading to myocardial lipotoxicity and atherosclerosis. Therefore, targeting lipid transport receptors in endothelial cells is regarded as a highly promising new strategy for intervening in cardiovascular metabolic diseases [56]. The potent influence of hepatically‐derived APOC3 and endothelial LPL on myocardial lipid supply underscores that cardiac metabolism cannot be viewed in isolation, but must be considered within a systemic framework.

#### 2.5.3 APOC3: A Multifaceted Driver of Diabetic CVD

APOC3 has been identified as a key driver of diabetes‐associated CVD [12]. It participates in pathology through multiple mechanisms: inhibiting LPL activity and interfering with lipoprotein‐receptor interactions to impede TRL clearance; modifying HDL and LDL structure and function, impairing HDL‐mediated cholesterol efflux while enhancing LDL's atherogenicity; and directly activating inflammatory pathways and impairing pancreatic β‐cell function, establishing a vicious cycle of metabolic dysfunction, inflammation, and vascular injury [12]. Clinically, plasma APOC3 levels > 15 mg/dL are an independent cardiovascular risk factor in diabetic patients [6].

#### 2.5.4 VEGFB: Endogenous Protective Factors

In contrast to pathogenic factors like APOC3, certain endogenous factors exert cardioprotective effects on cardiac metabolism. For instance, cardiomyocyte‐specific overexpression of vascular endothelial growth factor B (VEGFB) has been shown to inhibit coronary LPL activity through multiple mechanisms. This reduces the accumulation of toxic lipid intermediates in the myocardium and ultimately enhances cardiac insulin sensitivity. These findings highlight the role of growth factors as key regulators in coordinating cardiac “vascular function” and “metabolic health,” providing a novel targeting strategy for the treatment of diabetic cardiomyopathy [57].

#### 2.5.5 ApoB and the ApoB/ApoA‐1 Ratio

ApoB, as the structural component of all atherogenic lipoproteins (VLDL, IDL, LDL, Lp(a)), has a plasma concentration that directly reflects the total burden of circulating atherogenic particles [13, 58]. Substantial evidence indicates ApoB provides superior cardiovascular risk prediction compared to LDL‐C, serving as an independent predictor of major adverse cardiovascular events (MACE) in ST‐segment elevation myocardial infarction (STEMI) patients [13, 58, 59]. The ApoB/ApoA‐1 ratio is regarded as a superior “lipid balance indicator.” It is significantly elevated in patients with calcific aortic valve disease (CAVD) and identified as an independent risk factor [28]. The superior predictive power of ApoB and the ApoB/ApoA‐1 ratio highlights the need to go beyond traditional cholesterol metrics to quantify atherogenic particle number and balance, which is vital for identifying residual risk in patients with normal LDL‐C levels.

#### 2.5.6 PCSK9 and Emerging Apolipoprotein Panels

Proprotein convertase subtilisin/kexin type 9 (PCSK9) functions as a central systemic regulator. By promoting hepatic LDL receptor (LDLR) degradation, it reduces LDL‐C clearance and elevates plasma levels [60]. Gain‐of‐function PCSK9 mutations increase coronary heart disease risk, while PCSK9 inhibitors (e.g., evolocumab) potently lower ApoB and LDL‐C, representing a breakthrough in atherosclerotic cardiovascular disease (ASCVD) management [58, 60]. Beyond classical apolipoproteins, a multi‐apolipoprotein panel (ApoA‐I, ApoA‐IV, ApoC‐I, ApoD, ApoH, ApoJ) is independently associated with cardiovascular events, explaining 64% of case‐control differences and offering new perspective for assessing residual risk with normal LDL‐C [33]. Future research should extend beyond apolipoprotein concentrations to investigate their functional modifications (e.g., of APOC3 or ApoA‐I) under pathological states, as these functional changes may hold greater pathophysiological significance than abundance alone.

In summary, apolipoproteins play a central role in maintaining systemic lipid homeostasis and cardiac health. Their understanding has enhanced knowledge of CVD pathogenesis and propelled targeted therapies like APOC3 and PCSK9 inhibitors.

### 2.6 Integrated View: The Lipotoxic Cycle

**FIGURE 2:**
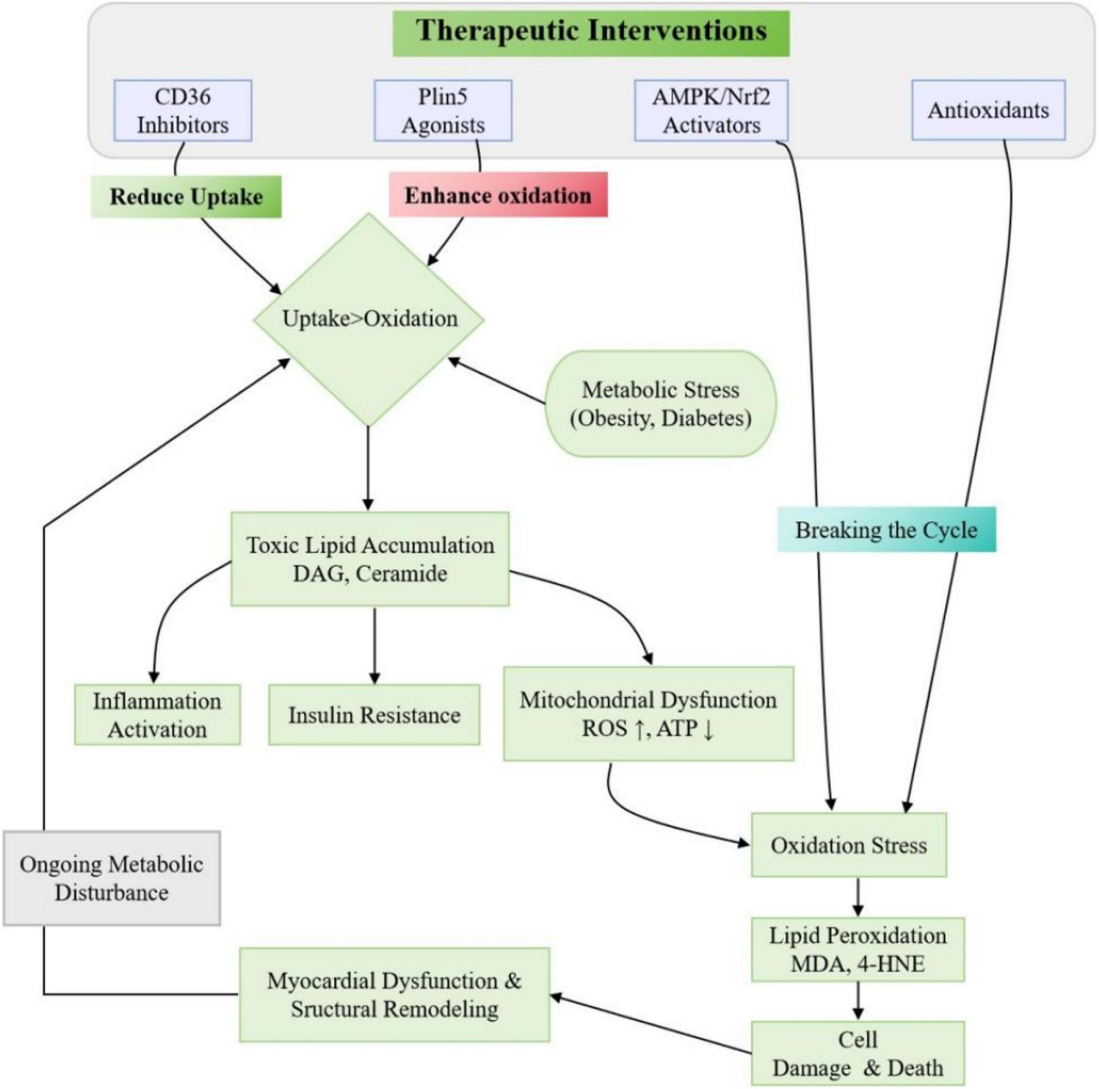
The lipotoxic cycle: a unifying pathological driver in cardiovascular diseases. This model illustrates the self‐reinforcing vicious cycle that propagates myocardial injury across various CVDs. The cycle is initiated by an imbalance between lipid uptake (e.g., via CD36) and oxidation capacity, leading to the accumulation of toxic lipid species (e.g., DAG, ceramides). These lipids trigger mitochondrial dysfunction (ROS overproduction, reduced ATP), insulin resistance, and inflammatory activation. Subsequent oxidative stress and lipid peroxidation generate reactive aldehydes (MDA, 4‐HNE), which inflict direct macromolecular damage and promote cell death, ultimately driving myocardial dysfunction and remodeling. This remodeling further exacerbates the initial metabolic imbalance, perpetuating the cycle. Key molecular nodes (e.g., CD36, Plin5, ROS) represent potential therapeutic targets for intervention.

Dysregulation of the aforementioned core network often converges on a self‐reinforcing pathological cascade known as the lipotoxic cycle (Figure 2). The cycle is typically initiated by an imbalance between lipid uptake (primarily via CD36) and mitochondrial oxidation capacity, leading to the cytosolic accumulation of toxic lipid intermediates like DAG and ceramides. These species instigate insulin resistance, inhibit SERCA2a activity, and promote endoplasmic reticulum stress. Concurrently, lipid overload and ceramides induce mitochondrial dysfunction, resulting in excessive reactive oxygen species (ROS) production. This oxidative stress, in turn, fuels lipid peroxidation, generating highly reactive aldehydes (e.g., malondialdehyde [MDA], 4‐hydroxynonenal [4‐HNE]) that cause direct cellular damage and activate pro‐inflammatory and pro‐apoptotic pathways. This cascade ultimately leads to cardiomyocyte dysfunction and death, which further exacerbates the initial metabolic disturbance, thereby perpetuating the cycle (Figure 2). While this model provides a powerful unifying framework, the relative contribution of specific nodes (e.g., CD36 vs. mitochondrial defects) likely varies with disease etiology and stage, demanding precision medicine approaches for effective intervention. Furthermore, the success of therapies targeting this cycle will be critically dependent on the timing of intervention, as strategies effective in early stages may be insufficient once widespread oxidative damage and cell death pathways are fully engaged.

## 3 Pathological Mechanisms in Cardiovascular Diseases

### 3.1 The Centrality of Lipotoxicity in CVD Pathogenesis

The dysregulation of cardiac lipid metabolism and the ensuing lipotoxic cycle, as detailed in Chapter 2, serve as a common pathological underpinning for a wide spectrum of cardiovascular diseases. While the core mechanism of lipid overload, oxidative stress, and cellular dysfunction remains consistent, its specific manifestations and relative contributions of different molecular nodes vary significantly across disease contexts. This chapter delineates how the lipotoxic cycle drives pathogenesis in major CVDs, and further explores its role in less canonical conditions induced by drugs, environmental factors, and developmental programming.

### 3.2 Atherosclerosis: Lipid‐Driven Inflammation and Plaque Formation

#### 3.2.1 Foam Cell Formation and Plin2 Dynamics

Macrophage‐derived foam cells drives atherosclerosis. After phagocytosis of oxidized LDL (oxLDL) uptake, macrophages accumulate cholesteryl esters within cytoplasmic LDs. Plin2 stabilizes LDs and promotes lipid retention by inhibiting nCEH and ABCA1‐mediated cholesterol efflux [20, 33, 43]. Plin2 also enhance CD36 expression, creating a self‐amplifying cycle of increased oxLDL uptake [33].

#### 3.2.2 Atherogenic Lipoproteins and Inflammatory Cascades

APOC3‐carrying LDL binds vascular proteoglycans and promotes inflammation, accelerating cholesterol deposition [12]. The ApoB/ApoA‐1 ratio reflects the imbalance between pro‐ and anti‐atherogenic forces. It predicts coronary artery disease and calcific aortic valve disease [28].

The interplay between lipid signaling and inflammatory responses further establishes a vicious cycle in atherosclerosis [61]. Metabolic disorders induced by atherosclerotic diets are far more than the classic lipoprotein abnormalities. Integrated metabolomics and lipidomics analysis revealed that such diets could trigger complex metabolic disturbances within the myocardium, including metabolic disorders of sulfur‐containing amino acids and inhibition of stearoyl‐CoA desaturase 1 (SCD1) activity, thereby leading to the accumulation of low‐saturation toxic TG in the heart myocardium and driving myocardial metabolic imbalance and dysfunction [62]. While CD36 serves as the primary receptor for macrophage recognition and uptake of oxLDL, its systemic deficiency may paradoxically accelerate atherosclerosis by driving chronic subclinical inflammation through eicosanoid imbalance and CXCR4/CCR2‐mediated immune cell recruitment [43]. Glycosphingolipids like lactosylceramide promote plaque development by disrupting lipid raft integrity and activating inflammatory pathways [21].

#### 3.2.3 Bioactive Phospholipids and Oxidized Cholesterol Toxicity

Lysophospholipids have dual roles in atherosclerosis. Lysophosphatidic acid (LPA) drives inflammation and plaque instability. Sphingosine 1‐phosphate (S1P) mobilize stem cells and protect cardiomyocytes. Targeting these pathways may offer therapeutic potential [63].

Regarding oxidized cholesterol toxicity, 7‐ketocholesterol—a primary toxic component of oxLDL—induces lipid metabolic reprogramming in cardiomyocytes by inhibiting the mevalonate pathway while activating phospholipase A2 (PLA2) and sterol O‐acyltransferase (SOAT). This reprogramming manifests as cholesteryl ester accumulation, reduced TG, and elevated lysophospholipids, potentially representing a key mechanism through which 7‐ketocholesterol participates in CVD pathogenesis [64].

The transition of Plin2 from a simple lipid storage regulator in cardiomyocytes to a central driver of foam cell formation in macrophages exemplifies the cell‐type‐specific functions of lipid metabolic proteins, highlighting the need for cell‐specific therapeutic targeting in atherosclerosis.

### 3.3 Heart Failure: Metabolic Remodeling Across the Ejection Fraction Spectrum

#### 3.3.1 HFrEF: Progressive Substrate Shift and Energy Deficiency

Heart failure with reduced ejection fraction (HFrEF) demonstrates stage‐specific metabolic alterations. Early stages maintain FAO. Advanced disease shows mitochondrial dysfunction and FAO decline [5, 46]. A “lipid paradox” emerges: high plasma FFA coexist with defective mitochondrial β‐oxidation [65]. Ketone oxidation compensates via SCOT and BDH1 upregulation [65, 66]. The liver synthesizes ketones via HMGCS2/HMGCL. The heart oxidizes them via SCOT/BDH1 [65]. Ketones serve as backup fuel under stress. Heart‐specific SCOT or BDH1 knockout worsens stress response [67]. The AMPK/PGC‐1α/PPARα axis activates but cannot reverse late‐stage energy deficiency [46, 47].

#### 3.3.2 HFpEF: Lipotoxicity and Diastolic Dysfunction

Lipotoxicity drives HFpEF, especially the cardiometabolic subtype. Dysfunctional epicardial adipose tissue releases FFAs and inflammatory factors. CD36‐mediated lipid uptake exceeds oxidation capacity [19, 42]. Toxic lipids accumulate. This causes diastolic dysfunction via insulin resistance, oxidative stress, and calcium handling defects [1, 19, 68]. The ketone oxidation shift in HFpEF is weaker than in HFrEF, likely due to insulin resistance impairing ketone utilization [22]. In addition to improving insulin sensitivity, directly targeting the lipid composition of the heart has also been proven to be an effective strategy for enhancing the diastolic function of HFpEF. Studies have shown that the liver X receptor (LXR) agonist AZ876 can significantly alleviate subendocardial fibrosis and diastolic dysfunction in mouse models by endogenous reprogramming of the cardiac lipid profile, upregulating polyunsaturated fatty acid (PUFAs) levels and inhibiting inflammation. This provides a new lipid remodel‐based approach for the treatment of HFpEF [69].

#### 3.3.3 Therapeutic Modulation and Neuro‐Metabolic Interactions

Drugs can modulate HF through lipid metabolism. Sacubitril/valsartan promotes lipolysis and reduces lipid deposition [23]. Treating metabolic disorders in comorbid conditions (e.g., hypertension) is also important. In a spontaneously hypertensive rat model, the fatty acid amide hydrolase inhibitor URB597 activates the endogenous cannabinoid system, lowering blood pressure while enhancing the uptake and esterification of long‐chain fatty acids by the myocardium, thereby improving lipid metabolism disorders. However, it failed to reverse left ventricular hypertrophy, suggesting that for such complex conditions, a combined intervention strategy may be needed to achieve comprehensive morphological and metabolic repair [70]. Specific pathways like the Mib2‐Runx2‐Hmgcs2 axis have been identified as regulators of myocardial lipid accumulation in HFpEF [71].

The distinct metabolic phenotypes between HFrEF and HFpEF suggest that metabolic therapies need to be tailored to the specific heart failure phenotype. Notably, beyond HF subtypes, drug action pathways also exhibit diversity. For instance, in addition to regulating the AMPK/PGC‐1α/PPARα axis, metformin has been confirmed to inhibit the HIF‐1α/PPAR‐γ pathway. This effectively reduces myocardial hypertrophy, improves left ventricular function in high‐fat diet models, and significantly decreases cardiomyocyte apoptosis by 52%, providing a new mechanistic basis for its clinical application in obesity‐related myocardial diseases [55].

In addition to direct drug intervention in metabolic pathways, neuro‐metabolic interactions have also attracted increasing attention. In the chronic heart failure model, piloside C combined with electroacupuncture stimulation can improve myocardial lipid metabolism by regulating the IL‐6/JAK1/STAT3 signaling in cardiac glial cells, reduce FABP4‐mediated lipid uptake and alleviate lipid toxicity. Meanwhile, this model reveals the multi‐organ linkage phenomenon of pericardial fat, liver and myocardial lipid accumulation, emphasizing the integrity of systemic metabolic disorders in heart failure [72].

### 3.4 Diabetic and Obesity Cardiomyopathy: Metabolic Disorder and Myocardial Lipotoxicity

#### 3.4.1 Initiation by Lipid Metabolic Imbalance

Type 1 diabetes (T1D) cardiomyopathy begins with insulin deficiency. Glucose uptake drops. The heart over‐relies on fatty acids. CD36‐mediated uptake increases. PPARα‐driven oxidation becomes imbalanced. Lipid hydrolysis impairs. Severe lipid accumulation and toxicity result [73].

Diabetic cardiomyopathy originates from disrupted lipid homeostasis. Persistent CD36 membrane translocation and upregulated expression in cardiomyocytes lead to fatty acid uptake that far exceeds oxidative capacity [10, 16]. Systemic factors like elevated APOC3 levels further exacerbate myocardial lipid exposure by impairing triglyceride‐rich lipoprotein clearance [12]. This elevated lipolysis may involve alterations in phosphatase‐mediated signaling pathways, as demonstrated by okadaic acid‐induced sustained perilipin phosphorylation and uncontrolled lipolysis in adipocytes [40]. Excess fatty acids are converted to TG, but when storage capacity is exceeded, toxic intermediates including DAGs and ceramides accumulate [16, 17].

#### 3.4.2 Molecular Mechanisms of Lipotoxicity

Toxic lipids impair function through distinct pathways. Effects are dose‐ and time‐dependent. In H9c2 cells, low‐dose palmitic acid (≤ 0.25 mM, 4 h) enhances mitochondrial respiration. High‐dose (≥ 0.5 mM, 24 h) triggers damage, stress, and apoptosis Saturated fatty acids have “double‐edged sword” effects. Defining toxicity thresholds is crucial [74]. Ceramides activate specific PKC isoforms, reducing contractile sensitivity to Ca^2+^ and inducing insulin resistance, while DAGs contribute to dysfunction by inhibiting IRS1 phosphorylation and SERCA2a activity [16]. In addition to ceramides and DAGs, protein palmitoylation, a post‐translational modification, has also been confirmed to be a core link connecting lipotoxicity and insulin resistance. Under high‐fat conditions, excessive palmitoylation of key proteins (such as CD36 and GLUT4) leads to abnormal membrane localization and functional disorders, directly impounding insulin signaling. Therefore, targeting palmitoylating/depalmitoylating enzymes has emerged as an emerging strategic direction for improving insulin resistance in diabetic hearts [75]. Accumulated ceramides directly suppress the AMPK/PGC‐1α/PPARα axis, further weakening mitochondrial oxidative capacity [16].

#### 3.4.3 Mitochondrial Dysfunction and Systemic Involvement

Mitochondrial dysfunction and oxidative stress form a vicious cycle. FAO overload triggers ROS. Toxic lipids damage the electron transport chain. Abnormal cardiolipin remodeling reduces respiratory efficiency [29]. Other factors include FGF21 resistance, miRNA dysregulation (miR‐320a), autophagy defects, and dysfunctional epicardial adipose tissue [14, 27, 68, 76, 77]. Among the numerous influencing factors, the role of endogenous protective factors cannot be ignored. Pigment epithelium‐derived factor (PEDF) has been confirmed to be a key molecule in improving lipid toxicity in diabetic cardiomyopathy. It exerts protective effects through multiple pathways: regulating cardiac energy metabolism, activating the Nrf2/HO‐1 pathway to enhance antioxidant capacity, and adjusting the balance of apoptotic proteins to reduce cardiomyocyte death, ultimately reversing cardiac remodeling and improving function [78]. The multifaceted nature of diabetic cardiomyopathy supports the potential of multi‐level interventional strategies rather than single‐target approaches.

### 3.5 Myocardial I/R Injury: Acute Metabolic Crisis and Reprogramming

#### 3.5.1 Metabolic Reprogramming During I/R

Myocardial I/R injury involves metabolic reprogramming and ionic imbalance. Ischemia interrupts oxygen supply. β‐oxidation fails. ATP drops. Sympathetic activation accelerates lipolysis. Circulating FFAs rise [24]. However, upon oxygen restoration during early reperfusion, the myocardial metabolic state fails to normalize immediately. The influx of high‐concentration FFAs at this stage may induce an “oxygen‐wasting” phenomenon—increased oxygen consumption without effective conversion to ATP production—thereby exacerbating oxidative stress and cellular injury [24].

#### 3.5.2 MicroRNA Dynamics and Endogenous Protection

miRNAs show “dual‐phase actions” during I/R. Early ischemia (< 6 h): miR‐27a‐3p activates glycolysis via TRAF5 targeting. ATP production maintains. Later ischemia (> 6 h): miR‐125b suppresses HK2 and LDHA. Glycolysis fails. Fatty acid uptake increases. Lipotoxicity and necrosis worsen [27].

Endogenous protective mechanisms play crucial roles in countering I/R injury. The LDs‐coated protein Plin5 optimizes directed FFA transport from storage sites to mitochondria for oxidation by suppressing excessive lipolysis and maintaining LDs‐mitochondria physical interaction. This reduces cytosolic FFA accumulation and its ensuing lipotoxicity while effectively constraining mitochondrial ROS burst, ultimately significantly limiting infarct size [2, 9]. Studies confirm that Plin5 overexpression can reduce infarct area by up to 58% [2].

The temporal regulation of metabolic pathways during I/R highlights the importance of timing in therapeutic interventions, where strategies effective in early ischemia may become detrimental during reperfusion. When seeking intervention strategies against MIR injury, traditional Chinese medicine compound prescriptions with multi‐target characteristics have shown potential. For instance, Xin‐Ji‐Er‐Kang protects against MIR injury in mice via a dual mechanism. It inhibits inflammatory responses and restores disordered glycerophospholipid and glyceride metabolism, up‐regulating beneficial membrane phospholipids and down‐regulating harmful lipids. This comprehensively improves cardiac function and pathological remodeling [79]. However, the complex composition of herbal formulations presents challenges for mechanistic dissection, standardization, and regulatory approval, limiting their current translational potential [31].

Beyond metabolic reprogramming, maintaining ion homeostasis under hypoxia is equally crucial. Studies have shown that in hypoxic cardiomyocytes, hypoxia‐inducible factor 2α (HIF‐2α)—rather than HIF‐1α—effectively preserves intracellular Na^+^/K^+^ homeostasis and electrophysiological function by specifically upregulating the transcription and membrane localization of the Na,K‐ATPase α2 subunit (α2‐NKA). This reveals a novel target distinct from metabolic pathways for targeted intervention in ischemic heart disease [80].

### 3.6 Genetic and Rare Metabolic Cardiomyopathies

#### 3.6.1 Fabry Disease and Lysosomal Storage Disorders

Fabry Disease. Fabry disease is an X‐linked lysosomal storage disorder. α‐Galactosidase A deficiency causes Gb3 accumulation in cardiomyocytes, endothelium, and conduction tissue. Result: left ventricular hypertrophy, arrhythmias, and heart failure [81]. Later‐onset variants often show cardiac dominance. Early enzyme replacement or chaperone therapy can stabilize or reverse dysfunction. Genetic screening is important in unexplained hypertrophic cardiomyopathy [81].

#### 3.6.2 Other Rare Disorders

Additional rare conditions with prominent cardiac lipid metabolic disturbances include [1]: **Danon disease**: LAMP2 deficiency causing autophagic vacuolar cardiomyopathy with massive glycogen and lipid accumulation [81] [2]. **PRKAG2 cardiomyopathy**: AMPK γ2 subunit mutations causing glycogen storage cardiomyopathy with conduction abnormalities [3]. **Carnitine deficiency disorders**: Primary or secondary carnitine deficiency impairing long‐chain fatty acid transport into mitochondria. These monogenic disorders, while individually rare, provide critical insights into the consequences of specific metabolic pathway disruptions and underscore the value of genetic testing in patients with atypical cardiomyopathy presentations.

### 3.7 Drug‐Induced and Environmentally Triggered Cardiomyopathy

#### 3.7.1 Drug‐Induced Cardiotoxicity

Doxorubicin causes cardiotoxicity via metabolic disturbances. It suppresses PPARα in a dose‐dependent manner. Fatty acid transport proteins and β‐oxidation enzymes downregulate. Lipid synthesis pathways activate aberrantly [26]. Another significant pathway involves the specific upregulation of FABP4 and Slc27a2/FATP2 gene expression, driving excessive fatty acid uptake [82]. Notably, the ALDH2 agonist Alda‐1 can effectively mitigate doxorubicin‐induced acute myocardial injury by reversing Slc27a2 overexpression [82] Table 1.

**TABLE 1:** Alterations in cardiac lipid metabolism across diverse pathological conditions.

Pathological condition	Key regulators/Pathways	Refs.
Drug‐induced Cardiotoxicity (e.g., Doxorubicin)	FATP2/Slc27a2, PPARα, ALDH2, P‐gp	[25, 26, 82]
Environmental Toxins (e.g., PM2.5 Exposure)	FGFR1 (Methylation), AMPK, PGC‐1α	[83]
Developmental Programming (e.g., Maternal Obesity/Alcohol)	PPARα, MCAD, Folate Cycle	[84, 85]
Oxidized Cholesterol Toxicity (e.g., 7‐Ketocholesterol)	Mevalonate Pathway, PLA2, SOAT	[64]
Progenitor Cell‐Driven Fibrosis	PPARγ, Akt, Gsk3β, FABP4	[86]
Alcoholic Cardiomyopathy	Plin5, LDs‐Mitochondria Contact	[37]
Fabry Cardiomyopathy	α‐Galactosidase A, Gb3 Accumulation, Lyso‐Gb3	[81]
Danon Disease	LAMP2, Autophagic Vacuolar Accumulation	[81]
Carnitine Deficiency	CPT1, Long‐Chain Fatty Acid Transport	[81]

Critical Evaluation of Experimental Findings. While these preclinical studies provide valuable mechanistic insights, several limitations warrant cautious interpretation. Most experiments utilize acute, high‐dose exposure models that may not fully recapitulate the chronic, low‐dose exposure in clinical chemotherapy regimens. Additionally, species differences in drug metabolism (e.g., higher doxorubicin conversion to cardiotoxic metabolites in rodents) may exaggerate cardiac toxicity compared to human patients [31]. Furthermore, the efficacy of ALDH2 agonists demonstrated in rodent models requires validation in human‐relevant systems, given the different ALDH2 variant frequencies across populations.

#### 3.7.2 Environmental Toxins

The impact of environmental factors on cardiac metabolism warrants equal attention. Exposure to fine particulate matter (PM2.5) can induce methylation of the FGFR1 promoter region, suppressing its expression and subsequently downregulating the AMPK‐PGC1α signaling pathway. This sequence of events leads to abnormal myocardial mitochondrial function and disordered lipid metabolism, ultimately causing cardiac dysfunction [83] (Table 1).

#### 3.7.3 Alcoholic Cardiomyopathy

Alcoholic cardiomyopathy shows an adaptive mechanism. Ethanol downregulates Plin5. LDs‐mitochondria contacts reduce. FA flux blocks. LDs accumulate. But this prevents β‐oxidation overload, ROS burst, and mitochondrial damage. Apoptosis and inflammation decrease. Artificially restoring LDs‐mitochondria contacts conversely exacerbates lipotoxicity, demonstrating that the functional outcome of organelle interaction can undergo a fundamental shift from protective to detrimental depending on the specific stress context [37] (Table 1).

### 3.8 Developmental Origins of Cardiac Metabolic Risk

#### 3.8.1 Maternal and Fetal Origins

Maternal metabolism during pregnancy affects offspring cardiac lipid metabolism. Maternal obesity alters lipid transport and remodels the fetal lipidome. Sexual dimorphism exists. Female fetal hearts are more sensitive. They show more lipid alterations. Male fetuses compensate by early PPARα activation [84]. Consistently, during fetal development, premature exposure to exogenous lipids (e.g., lipid emulsions in parenteral nutrition) has been shown to induce complex metabolic reprogramming in fetal sheep cardiomyocytes. On one hand, it upregulates genes like CD36 (by 54%) and CPT1A (by 291%), promoting long‐ and very long‐chain fatty acid uptake and LDs formation; on the other hand, it inhibits maximum mitochondrial oxygen consumption rate (by 24%), an effect exacerbated by palmitate treatment (40% decrease in OCR). This ultimately leads to myocardial metabolic dysfunction, highlighting the profound impact of the lipid environment in early development on cardiac metabolic programming [87] (Table 1).

#### 3.8.2 Paternal and Intergenerational Inheritance

Not only the maternal environment, but also the diet of the father can influence the cardiometabolic health of the offspring through intergenerational programming. Studies have shown that high carbohydrate intake by parents can alter the systemic lipid metabolism networks of both offspring and grandchildren, leading to specific “rerouting” of lipids between tissues (such as from the heart to the central nervous system), which provides a brand‐new mechanistic perspective for understanding the intergenerational genetic risk of cardiovascular metabolic diseases [88] (Table 1).

### 3.9 Emerging Concepts: Non‐Canonical Mechanisms and Systemic Regulation

#### 3.9.1 The Heart as a Systemic Metabolic Regulator

The heart regulates systemic lipid homeostasis beyond energy consumption. The Snail transcription factor in Drosophila hearts regulates lipolysis and lipogenesis genes in fat bodies. This non‐cell‐autonomous mechanism maintains systemic lipid balance. Loss of cardiac Snail function leads to systemic obesity, while its overexpression promotes a systemic lean phenotype. This reveals the heart's novel function as a key “metabolic regulatory organ” that remotely coordinates systemic energy homeostasis via secreted factors or neural signals [89].

#### 3.9.2 ER Stress and Lipotoxicity: A Vicious Cycle

ER stress and lipotoxicity form a core pathological axis. ER stress disrupts lipid metabolism via unfolded protein response (UPR). This worsens lipotoxicity. Conversely, lipotoxicity induces ER stress. This vicious cycle drives ischemic heart disease and heart failure. Targeting ER stress has become a promising therapeutic direction [90].

#### 3.9.3 Progenitor Cell Differentiation and Metabolic Fibrosis

Concerning the cellular origins of cardiac fibrosis, recent research reveals that under conditions of hyperlipidemia combined with hypertension, non‐bone marrow‐derived CD34^+^ progenitor cells can differentiate into FABP4^+^ fibroblasts via the PPARγ/Akt/glycogen synthase kinase 3β (Gsk3β) pathway. This cellular subpopulation possesses a strong capacity for triglyceride accumulation and accelerates fibrotic progression by promoting cardiac lipotoxicity, providing a novel cellular explanation for metabolic cardiac fibrosis [86].

Collectively, these diverse pathological conditions demonstrate that cardiac lipid metabolism constitutes a highly dynamic system exquisitely sensitive to environmental stimuli. Its adaptive reprogramming or functional compromise under various stresses profoundly influences the heart's transition from health to disease. Especially remarkable is the realization that certain alterations—such as reduced LDs‐mitochondria contacts in alcoholic cardiomyopathy—may represent protective compensatory mechanisms in specific contexts. This challenges the traditional view that promoting FAO is invariably beneficial and underscores the necessity of tailoring metabolic interventions to the precise pathological background. In‐depth investigation of these specialized pathological models provides an indispensable dimension for comprehensively understanding the complex regulatory network of cardiac metabolism.

## 4 Oxidative Stress and Lipid Peroxidation

### 4.1 ROS Generation and the Self‐Perpetuating Lipotoxic Cycle

In cardiac pathophysiology, oxidative stress and disrupted lipid metabolism form a self‐perpetuating vicious cycle that significantly accelerates cardiomyocyte damage. Disordered lipid metabolism establishes a critical foundation for ROS generation. When myocardial lipid uptake exceeds mitochondrial oxidative capacity, the excessive influx of fatty acids overloads the electron transport chain, triggering substantial production of ROS including superoxide anion (O_2_•^−^) [9, 17]. Notably, toxic lipids themselves possess direct pro‐oxidant effects—ceramides have been demonstrated to specifically inhibit mitochondrial respiratory chain complex III activity, increasing the probability of electron leakage [16].

#### 4.1.1 ROS‐Mediated Exacerbation of Lipid Metabolic Dysfunction

Correspondingly, excessive ROS further deteriorates lipid metabolic status. ROS can attack mitochondrial membrane structures and enzyme systems, inhibiting fatty acid β‐oxidation efficiency and causing more lipid intermediates to accumulate abnormally in the cytoplasm. More importantly, ROS directly attacks polyunsaturated fatty acids (PUFAs) in cell membranes and LDs, initiating the chain reaction of lipid peroxidation that generates highly reactive aldehyde toxic end products including MDA and 4‐HNE [9, 91, 92]. The intricate coupling between lipid overload and ROS generation creates a feed‐forward loop that is difficult to interrupt once established, emphasizing the importance of early intervention targeting both metabolic and oxidative components.

### 4.2 Lipid Peroxidation Products and Direct Cellular Damage

The pathological significance of lipid peroxidation extends beyond the consumption of PUFAs and membrane destruction to include the direct damaging effects of its secondary metabolites on cells.

#### 4.2.1 4‐HNE‐Mediated Protein Damage

4‐HNE is a major toxic messenger. Its electrophilicity forms Michael adducts with cysteine, histidine, and lysine. This alters protein structure and function [91, 92]. In the heart, 4‐HNE targets are widely distributed among key energy metabolic enzymes (e.g., α‐ketoglutarate dehydrogenase and electron transport chain complexes I and II), calcium‐handling proteins (e.g., SERCA2a), and stress signaling pathway components. While low concentrations can activate Nrf2‐mediated protective responses, sustained high‐level exposure shifts toward pro‐apoptotic JNK and p38 MAPK, ultimately inducing cardiomyocyte death [91].

#### 4.2.2 MDA and Cross‐Linking Damage

MDA cross‐links proteins. It forms Schiff bases with amino groups. This causes protein aggregation and enzyme inactivation [93]. MDA also reacts with DNA to form mutagenic adducts. MDA levels serve as oxidative stress biomarkers in CVD [2, 26, 82]. In addition to serving as biomarkers, the levels of lipid peroxidation products and their precursors themselves are also modifiable. For instance, low‐dose PDE4 inhibitor Rolipran has been proven to reduce the unsaturated/saturated lipid ratio in the hearts of normal mice, inhibit lipid peroxidation (manifested as a decrease in MDA levels), and also regulate the physical properties of myocardial cell membranes and the secondary structure of proteins. This indicates that precisely regulating the molecular component environment of the heart through drugs is a potential approach to alleviating oxidative damage [94].

#### 4.2.3 Convergence on Multiple Cell Death Pathways

Lipid peroxidation products disrupt membrane integrity. They open mitochondrial permeability transition pore (mPTP). They activate apoptosis, necrosis, and ferroptosis [95]. Lipid peroxidation triggers ferroptosis in cardiac fibroblasts during fibrosis [91]. This mechanism extends to cancer therapy. Medium‐chain fatty acids (lauric acid, caprylic acid) upregulate CD36 and ACSL4 in cancer cells. This promotes ω‐6 PUFA uptake and conversion to peroxidation substrates. Cancer cells become more sensitive to ferroptosis inducers [96]. Peroxidation products also activate mechanosensitive ion channels (Piezo1), worsening injury [95, 97].

The diverse reactivity of different lipid peroxidation products—with 4‐HNE acting mainly through protein adduction and MDA through cross‐linking—suggests they may damage cells through complementary mechanisms, making combined clearance strategies potentially more effective than targeting single species.

### 4.3 Integrated Endogenous Antioxidant Defense Systems

Faced with the persistent threat of oxidative stress and lipid peroxidation, cardiomyocytes activate a multi‐layered endogenous antioxidant defense system.

#### 4.3.1 Nrf2: The Master Regulator of Inducible Defenses

Nrf2 regulates antioxidant defense. Under homeostasis, Nrf2 binds Kelch‐like ECH‐associated protein 1 (Keap1) and targeted for ubiquitin‐mediated degradation. Oxidative stress or electrophiles (e.g., 4‐HNE), dissociate this complex. Nrf2 moves to the nucleus. It transcribes cytoprotective genes: NAD(P)H quinone dehydrogenase 1 (NQO1), heme oxygenase‐1 (HO‐1), and the glutamate‐cysteine ligase catalytic subunit (GCLC) [15]. In DCM, Nrf2 activation demonstrates multidimensional cardioprotective effects: it effectively clears ROS and reduces levels of lipid peroxidation products like MDA, while simultaneously activating the AMPK/SIRT1/PGC‐1α branch of the AMPK/PGC‐1α/PPARα axis, thereby improving lipid metabolism and indirectly repairing insulin signaling damaged by lipotoxicity [15].

#### 4.3.2 GPX4: The Core Executor Against Ferroptosis

GPX4 defends against lipid peroxidation and suppresses ferroptosis. It uses GSH to reduce phospholipid hydroperoxides. This blocks peroxidation chain reactions [95, 97]. During cardiac fibrosis progression, the activation of cardiac fibroblasts is accompanied by decreased GPX4 activity, exacerbating lipid peroxidation and inducing ferroptosis. Research indicates that downregulation of glutathione S‐transferase Mu 1 (GSTM1) promotes cardiac fibroblast activation through mechanisms closely associated with ferroptosis. Conversely, GSTM1 overexpression enhances antioxidant capacity by activating the GSH/ROS/STAT3/GPX4 signaling axis, inhibiting lipid peroxidation and ferroptosis, and significantly alleviating post‐infarction cardiac fibrosis while improving cardiac function [97].

#### 4.3.3 Additional Defense Mechanisms and Regulatory Nodes

Plin5 has dual protective effects in the oxidative stress‐lipotoxicity cycle. It guides fatty acids to oxidation via LDs‐mitochondria contacts. It enhances ROS clearance. Plin5 overexpression reduces mitochondrial ROS by 42% and MDA by 58% in DCM [2, 9].

Concurrently, nuclear factor erythroid 2‐related factor 2 (Nrf2) also activates in DCM., It upregulates the expression of antioxidant enzymes including superoxide dismutase (SOD) and glutathione peroxidase (GPx), effectively clearing excess ROS [15]. CD36 deficiency reduces plasmalogens. This shows membrane components matter for antioxidant defense [43].

The hierarchical organization of the antioxidant defense system—with Nrf2 controlling inducible responses and enzymes like GPX4 executing specific detoxification reactions—provides multiple regulatory nodes for therapeutic intervention, but also reveals vulnerabilities when any component is compromised.

## 5 Biomarkers and Clinical Translation

### 5.1 Circulating Biomarkers for Diagnosis and Risk Stratification

#### 5.1.1 Protein Biomarkers

The identification of LDs‐associated proteins in circulation provides novel insights into metabolic status. Circulating Plin2 levels demonstrate a strong positive correlation with coronary artery stenosis severity and plaque instability, establishing its potential as a real‐time indicator of atherosclerotic progression [6, 34]. In contrast, myocardial tissue Plin5 expression correlates positively with left ventricular ejection fraction recovery, serving as a favorable prognostic marker for functional improvement following ischemic injury [6]. The differential expression patterns of perilipin proteins in circulation versus myocardial tissue highlight their distinct clinical utilities—circulating levels reflecting systemic disease burden while tissue expression indicating local metabolic adaptation.

Plasma APOC3 levels exceeding 15 mg/dL establish a clear threshold for cardiovascular risk stratification in diabetic populations, with concentrations above this cutoff representing an independent risk factor for major adverse cardiac events [12]. Concurrently, elevated serum FGF21 levels in heart failure patients strongly associate with disease progression and poor outcomes, providing clinicians with a valuable tool for monitoring therapeutic response and disease trajectory [14]. The significant upregulation of Slc27a2/FATP2 mRNA in peripheral blood mononuclear cells of obese individuals, coupled with its correlation with established myocardial injury markers, positions this transporter as a promising circulating indicator of obesity‐driven lipotoxic cardiomyopathy [44].

The emergence of these protein biomarkers enables a more nuanced assessment of metabolic cardiovascular health, moving beyond traditional lipid panels to provide dynamic information about ongoing pathological processes (Table 2).

**TABLE 2:** Summary of therapeutic strategies targeting cardiac lipid metabolism.

Category	Representative agents/Targets	Core mechanism	Stage & advantages	Key challenges	Refs.
Gene & Protein Targeting	APOC3 Inhibitors (Volanesorsen, Plozasiran)	Reduces APOC3 synthesis, enhances LPL activity, lowers TG.	**Clinical (Ph III)**: Potent TG reduction; long‐acting (siRNA).	Injection‐site reactions; cost.	[12]
	PCSK9 Inhibitors (Evolocumab)	Blocks PCSK9, increases hepatic LDL‐C clearance.	**Approved**: Proven CV benefit; strong LDL‐C lowering.	Limited effect on lipotoxicity; cost.	[58, 60]
	CD36 Modulation (Partial knockdown)	Reduces myocardial fatty acid uptake.	**Preclinica**l: Targets lipotoxicity at its source.	Poor tissue specificity; potential systemic effects.	[10, 41, 98]
Metabolic Pathway Modulators	Plin5 Agonists/Gene Therapy (AAV9‐Plin5)	Enhances LDs‐mitochondria contact, optimizes FA flux & oxidation.	**Preclinical**: Improves metabolic efficiency & redox state.	Long‐term safety and delivery of gene therapy.	[2, 6, 9]
	SGLT2 Inhibitors (Empagliflozin, dapagliflozin)	Activates AMPK, inhibits CD36/ceramide, promotes metabolic health.	**Approved**: (HF/CKD)Cardio‐renal benefits beyond glucose control.	Exact cardiac mechanisms under investigation.	[16, 30]
	Nrf2 Activators (Sulforaphane)	Boosts endogenous antioxidant defenses, reduces oxidative stress.	**Early Clinical**: Breaks ROS‐lipotoxicity cycle.	Lack of large CV outcomes trials.	[15]
	AMPK Agonists (Metformin)	Activate the AMPK/PGC‐1α/PPARα pathway and improve lipid oxidation.	**Clinical Application**: Improve metabolism‐related atrial remodeling.	Limited efficacy in advanced heart failure.	[15, 47]
Natural Products & TCM	Flavonoids/Polyphenols (Puerarin, resveratrol)	Multi‐target: Activates AMPK/SIRT1, promotes FAO, anti‐inflammatory.	**Preclinical/Early Clinical**: Systemic modulation with good safety.	Low oral bioavailability; complex pharmacokinetics.	[7, 99, 100]
	TCM Formulae (Linggui Zhugan Decoction, Qifu Decoction)	Linggui Zhugan Decoction: Activates the Apelin/APJ pathway; Qifu Decoction: Regulates the metabolism of glycerophospholipids and sphingolipids.	**Clinical Evidence**: Holistic regulation for complex conditions.	Standardization and mechanistic clarification.	[101, 102, 103]
Gene & Cell Therapy	mRNA Therapy(Cardiac‐targeted P‐gp mRNA)	Transiently expresses efflux pumps in cardiomyocytes to protect from toxins.	**Preclinical**: Precise, transient protection (e.g., from chemo).	Myocardial targeting efficiency and safety of LNPs.	[25]
	miRNA Regulation (miR‐30c Mimics, Anti‐miR‐320a)	Activate fatty acid oxidation/inhibit excessive lipid uptake.	**Preclinical**: Precisely regulate metabolic pathways.	Targeting of in vivo delivery; off‐target effects.	[25, 27]
Novel Interventions	Time‐Restricted Eating	Entrains circadian rhythms of metabolic genes for optimal lipid handling.	**Clinical Research**: Non‐pharmacologic, scalable lifestyle intervention.	Long‐term patient adherence; individual variability.	[25, 104]
	Gut Microbiome Modulation(Prebiotics)	Alters microbial metabolites (e.g., SCFAs) to systemically influence cardiac metabolism.	**Early Clinical**: Novel “gut‐heart axis” target.	Strain‐specific effects; high inter‐individual variation.	[23]
**Advanced Metabolic Imaging**	PET (^18^F‐FTHA, ^18^F‐FBnTP)	Quantifies regional FAO and mitochondrial function.	**Clinical**: Non‐invasive metabolic.	Radiation exposure; specialized facilities.	[105]
	MRS (^1^H‐MRS, ^31^P‐MRS)	Direct quantification of myocardial lipids and energy status.	**Clinical/Research**: No radiation; direct metabolite quantification.	Limited spatial resolution; high‐field requirements.	[106]

#### 5.1.2 Lipid and Metabolite Biomarkers

In the realm of lipid biomarkers, the ApoB/ApoA‐1 ratio demonstrates exceptional predictive value. This metric, proven superior to traditional lipid parameters, accurately reflects the balance between pro‐atherogenic and anti‐atherogenic lipoproteins. Studies indicate that a ratio ≥ 0.7 indicates high risk, and when combined with clinical parameters like age and diabetes history, it shows excellent predictive performance for CAVD risk (AUC = 0.796) [28]. In HF assessment, plasma or myocardial ceramide species (e.g., CER 16:0) closely associate with the degree of cardiac functional impairment and mortality risk [29].

Mitochondrial function‐related cardiolipins, especially the reduction of CL 72:8, serve as important markers of HF progression [29, 107]. Furthermore, in patients with arrhythmogenic cardiomyopathy, plasma β‐hydroxybutyrate levels are significantly elevated and correlate with disease severity, reflecting characteristic metabolic remodeling [108]. In advanced HF, myocardial ketone body uptake capacity (expressed as βHB fractional extraction rate) positively correlates with the extent of left ventricular remodeling, serving as a valuable indicator of metabolic compensation and disease severity [66]. The emergence of specific lipid species and metabolic intermediates as biomarkers provides unprecedented insight into the metabolic state of the failing heart, moving beyond traditional functional assessments.

#### 5.1.3 Emerging Biomarker Strategies

Emerging biomarker strategies are overcoming the limitations of traditional single‐parameter approaches. A multi‐apolipoprotein panel comprising ApoA‐I, ApoA‐IV, ApoC‐I, ApoD, ApoH, and ApoJ has been found to be independently associated with cardiovascular events, collectively explaining 64% of case‐control differences. This approach is particularly suitable for assessing residual risk in populations with normal traditional LDL‐C levels, offering a new perspective for risk refinement [33].

For clinically complex conditions like HFpEF, where diagnostic value of single lipid level changes is limited, the pattern of their association with clinical parameters (such as left atrial volume index) differs fundamentally between patients and healthy populations. This shift in “association patterns” itself may become a novel functional biomarker for diagnosing HFpEF, representing a paradigm shift from static biomarker measurement to dynamic relationship analysis [109]. The integration of multiple biomarkers into clinically actionable panels, combined with machine learning approaches for pattern recognition, represents the next frontier in cardiovascular diagnostics, focusing on systems‐level understanding rather than isolated molecular changes.

### 5.2 Advanced Metabolic Imaging Technologies

Beyond circulating biomarkers, advanced imaging modalities enable non‐invasive assessment of myocardial metabolism in vivo, providing complementary tools for disease characterization and therapeutic monitoring [32, 105, 109].

#### 5.2.1 Positron Emission Tomography (PET)

PET using radiolabeled fatty acid analogs (e.g., ^18^F‐fluoro‐6‐thia‐heptadecanoic acid, ^18^F‐FTHA) allows quantification of regional myocardial fatty acid uptake and oxidation. In heart failure, PET imaging consistently demonstrates reduced FAO rates and enhanced glucose utilization, reflecting the metabolic shift characteristic of the failing heart. Additionally, PET with ^18^F‐fluorodeoxyglucose (FDG) can assess myocardial glucose metabolism, while novel tracers targeting specific pathways (e.g., ^18^F‐FBnTP for mitochondrial membrane potential) provide insights into mitochondrial function [105].

#### 5.2.2 Magnetic Resonance Spectroscopy (MRS)

Proton (^1^H) and phosphorus (^31^P) MRS enable direct, non‐invasive quantification of myocardial lipid content and high‐energy phosphate metabolism, respectively. ^1^H‐MRS can detect myocardial triglyceride accumulation (a hallmark of lipotoxicity) with high reproducibility, while ^31^P‐MRS measures phosphocreatine/ATP ratios, providing direct assessment of myocardial energy status. These techniques have demonstrated reduced phosphocreatine/ATP ratios in heart failure patients, correlating with disease severity and prognosis [106].

#### 5.2.3 Single‐Photon Emission Computed Tomography (SPECT)

While primarily used for perfusion imaging, SPECT with iodine‐123‐labeled fatty acid analogs (e.g., ^123^I‐BMIPP) can assess fatty acid metabolism. Reduced ^123^I‐BMIPP uptake relative to perfusion (“mismatch”) indicates metabolic impairment and has prognostic value in ischemic heart disease and cardiomyopathies [106, 110].

#### 5.2.4 Data‐Driven Modeling Approaches

Integration of imaging data with computational metabolic flux analysis and machine learning algorithms enables construction of patient‐specific metabolic models. These approaches can predict individual responses to metabolic interventions and identify optimal therapeutic strategies, representing a step toward precision metabolic medicine [106, 111].

#### 5.2.5 Clinical Integration and Challenges

While these imaging modalities offer unprecedented insights, several challenges limit their widespread clinical adoption. PET requires specialized radiochemistry facilities and exposes patients to ionizing radiation. MRS suffers from limited spatial resolution and signal‐to‐noise constraints, requiring high‐field magnets and specialized expertise. Standardization of protocols and establishment of normative databases across diverse populations remain priorities for clinical translation [106, 110, 111].

### 5.3 Targeted Therapeutic Strategies

The mechanistic understanding of cardiac lipid metabolism has unveiled a promising landscape of therapeutic targets. These strategies aim to intervene at different nodes of the regulatory network, from systemic lipid modulation to intracellular metabolic fine‐tuning. As summarized in Table 2, these approaches can be broadly categorized into several paradigms, each with distinct mechanisms, representative agents, and developmental stages.

#### 5.3.1 Diverse Therapeutic Paradigms

Current strategies include gene and protein‐targeted agents that directly silence pathogenic factors (e.g., APOC3 inhibitors) or enhance beneficial pathways (e.g., PCSK9 inhibitors); metabolic pathway modulators that reprogram myocardial substrate utilization and energy sensing (e.g., SGLT2 inhibitors, AMPK agonists); natural products and system‐regulating interventions that offer multi‐target, holistic effects through phytochemicals, traditional formulations, or lifestyle modifications; and emerging gene and cell therapy approaches that enable precise, long‐term or transient metabolic corrections.

Critical Evaluation of Natural Products and Traditional Medicine. Traditional Chinese medicine (TCM) formulations such as Linggui Zhugan Decoction and Qifu Decoction have demonstrated cardioprotective effects in preclinical models through multi‐target modulation of lipid metabolism [101, 102]. However, these studies face significant translational limitations: complex, variable compositions challenge mechanistic dissection and quality control; lack of standardized extraction protocols hinders reproducibility; and limited rigorous clinical trials in well‐defined patient populations restrict evidence‐based recommendations. While these approaches offer theoretical advantages in addressing metabolic network complexity, their clinical integration requires rigorous pharmaceutical standardization and prospective validation [31].

#### 5.3.2 Towards Combination and Personalization

The diversity of these strategies underscores a key future direction: the move beyond single‐target approaches. The success of interventions will likely depend on effectively combining agents that target complementary pathways (e.g., simultaneously enhancing oxidation and reducing toxic uptake) and on matching specific therapeutic mechanisms to well‐defined patient subgroups based on their predominant metabolic disturbances.

## 6 Challenges and Future Perspectives

The translation of mechanistic insights into clinical applications faces several interconnected challenges that must be addressed to realize the full potential of precision metabolic medicine.

### 6.1 Knowledge Gaps and Network Complexity

The regulatory networks underlying cardiac metabolic reprogramming remain incompletely defined. While key nodes (e.g., AMPK/PGC‐1α/PPARα axis, Plin5, CD36) have been identified, their dynamic interactions, feedback loops, and context‐dependent behaviors are not fully understood. This uncertainty poses significant challenges to the development of highly specific and targeted therapeutic strategies. For example, the dual role of PPARα activation—beneficial in some contexts but potentially harmful in others—highlights our limited ability to predict network‐level consequences of single‐node interventions. Future research must employ systems biology approaches, integrating multi‐omics data with computational modeling to construct predictive network models that can guide therapeutic design [112].

### 6.2 Species‐Specific Differences and Model Limitations

As noted in Chapter 3, important metabolic differences exist between commonly used animal models and humans. Rodents exhibit higher baseline FAO rates, distinct lipoprotein profiles (predominantly HDL vs. LDL in humans), and different cardiac regenerative capacities. These differences may limit the predictive value of preclinical findings for clinical outcomes. The development of human‐induced pluripotent stem cell‐derived cardiomyocyte models, organoid systems, and large animal models (e.g., pigs) that better recapitulate human cardiac metabolism will be essential for improving translational fidelity [99, 112].

### 6.3 Context‐Dependent Functions and Pleiotropy

Key regulators like Plin5 and CD36 exhibit context‐dependent functions that complicate therapeutic targeting. Plin5's shift from protective to potentially harmful in alcoholic cardiomyopathy, and CD36's dual roles in lipid uptake and immune regulation, necessitate precise understanding of molecular behavior across pathological contexts. Developing tissue‐specific, pathway‐selective modulators rather than blunt activation or inhibition strategies will be critical [37, 41].

### 6.4 Translational Barriers

Additional barriers include the lack of tissue‐specific delivery systems for gene therapies and RNA‐based interventions, the critical timing of therapeutic intervention (exemplified by PPARα‘s dual roles), and the need for standardized biomarker assays and validation in diverse populations. The complexity of natural product formulations and the challenges of mechanistic dissection for multi‐component therapies further complicate regulatory pathways [25, 51, 97, 112].

### 6.5 Future Directions

Advancements in several areas will address these challenges:

#### 6.5.1 Single‐Cell and Spatial Multi‐Omics

These technologies will resolve cardiac metabolic heterogeneity at unprecedented resolution, revealing cell‐type‐specific metabolic programs and their spatial organization within the myocardium [10, 22].

#### 6.5.2 Advanced Imaging and Machine Learning

Integration of metabolic imaging with machine learning algorithms will enable precise phenotyping, early detection of metabolic dysfunction, and prediction of therapeutic responses [32, 98, 99, 111].

#### 6.5.3 Precision Medicine Frameworks

The ultimate goal is to establish personalized treatment frameworks that match specific mechanisms—such as APOC3 inhibition for hypertriglyceridemia, Plin5 activation for oxidation defects, or CD36 modulation for lipotoxic cardiomyopathy—to well‐defined patient subgroups. This will require [1]: Metabolic phenotyping: Integration of circulating biomarkers, advanced imaging, and genomic profiling to classify patients by their predominant metabolic disturbances [2]. Biomarker‐guided therapy: Using dynamic biomarker panels (e.g., ceramide species, ApoB/ApoA‐1 ratio, circulating perilipins) to monitor treatment response and adjust interventions [3]. Adaptive trial designs: Clinical trials that allow real‐time modification based on metabolic biomarker responses, accelerating the identification of optimal therapies for specific phenotypes [32, 109].

#### 6.5.4 Novel Therapeutic Modalities

Emerging technologies offer unprecedented precision in metabolic intervention [1]: RNA‐based therapeutics: siRNA (e.g., APOC3 inhibitors) and antisense oligonucleotides enable durable, specific silencing of pathogenic factors. Cardiac‐targeted delivery systems using lipid nanoparticles (LNPs) modified with cardiac‐specific ligands are under active development [2, 25]. Gene editing: CRISPR‐Cas9 approaches for correcting monogenic metabolic disorders (e.g., PRKAG2 cardiomyopathy) or modulating regulatory nodes (e.g., enhancing Plin5 expression) hold transformative potential, though delivery specificity and off‐target effects remain challenges [3]. Mitochondrial transplantation: Emerging evidence suggests that transferring healthy mitochondria to metabolically compromised cardiomyocytes can restore oxidative capacity, offering a novel approach for advanced heart failure where endogenous mitochondrial function is severely compromised.

#### 6.5.5 Artificial Intelligence and Systems Medicine

Machine learning algorithms trained on multi‐omics datasets can identify non‐obvious patterns linking metabolic states to clinical outcomes. These approaches can [1]: Predict individual responses to metabolic interventions based on baseline metabolic profiles [2]. Identify novel therapeutic targets through network analysis of metabolic dysregulation [3]. Optimize combination therapy selection by modeling drug‐metabolism interactions [32, 99].

#### 6.5.6 Circadian and Chronotherapy Integration

Growing evidence indicates that cardiac metabolism exhibits circadian rhythmicity, with FAO rates varying across the day. Time‐restricted feeding and chronotherapy—aligning drug administration with metabolic cycles—represent low‐cost, scalable strategies to optimize metabolic health without pharmacological intervention [104]. Future studies should define optimal timing for metabolic interventions to maximize efficacy.

#### 6.5.7 Interorgan Communication Networks

The heart's metabolic state is profoundly influenced by systemic signals from adipose tissue, liver, skeletal muscle, and gut microbiota. Understanding these interorgan communication networks—mediated by circulating metabolites, extracellular vesicles, and neural signals—will enable holistic therapeutic approaches that address cardiac lipotoxicity at its systemic origins rather than treating the heart in isolation [86, 89].

#### 6.5.8 Overcoming Translational Barriers

Realizing these advances requires concerted efforts to [1]: Develop standardized assays for emerging biomarkers (e.g., specific ceramide species, modified apolipoproteins) [2]. Establish international consortia for harmonizing metabolic imaging protocols and creating normative databases [3]. Create regulatory pathways for complex, multi‐component therapeutics including traditional medicine formulations [4]. Foster interdisciplinary collaboration integrating basic science, clinical cardiology, imaging physics, computational biology, and bioengineering.

The path forward demands a paradigm shift from reactive, one‐size‐fits‐all approaches to proactive, precision metabolic medicine. By integrating deep mechanistic understanding with advanced technologies and rigorous clinical validation, we can transform the prevention and treatment of cardiovascular diseases through the lens of cardiac lipid metabolism.

## 7 Conclusion

Cardiac lipid metabolism represents a dynamic and precisely regulated system fundamental to myocardial energy homeostasis. This review has synthesized the core regulatory network governing this process, centered on key molecules and pathways including Plin5‐mediated LDs‐mitochondria crosstalk, CD36‐facilitated fatty acid uptake, APOC3‐driven systemic lipid modulation, and the energy‐sensing AMPK/PGC‐1α/PPARα axis. Disruption of this intricate balance initiates a self‐perpetuating lipotoxic cycle, characterized by toxic lipid accumulation, oxidative stress, and inflammatory activation, which serves as a unifying pathological mechanism across diverse cardiovascular diseases, from atherosclerosis and heart failure to diabetic cardiomyopathy.

The translational implications of these mechanistic insights are profound. These core regulatory components not only illuminate novel pathogenic pathways but also emerge as promising biomarkers and therapeutic targets. This is evidenced by the rapid development of targeted strategies, such as APOC3 inhibitors, PCSK9 inhibitors, SGLT2 inhibitors, and emerging Plin5‐based gene therapies. Advanced metabolic imaging technologies (PET, MRS, SPECT) now enable non‐invasive assessment of myocardial metabolism, complementing circulating biomarkers to provide comprehensive metabolic phenotyping.

Looking ahead, the full potential of targeting cardiac lipid metabolism will be realized through the integration of multi‐omics technologies, refined disease modeling, and the advancement of precision medicine paradigms. Such efforts will enable the transition from generalized approaches to personalized therapeutic strategies—matching specific metabolic interventions to well‐defined patient subgroups based on their unique metabolic disturbances. Overcoming current challenges through interdisciplinary collaboration and technological integration will be essential to forge a new path for the prevention and treatment of cardiovascular diseases, ultimately transforming cardiovascular medicine through the lens of metabolic health.

## Author Contributions

**Peiyun Xie** and **Qun Zeng:** Drafting the article, substantial contributions to conception and design. **Hongrui Wang:** Analysis and interpretation of data. **Meihua She:** Drafting the article and revising it critically for important intellectual content, Final approval of the version to be published.

## Conflicts of Interest

The authors declare no conflicts of interest.

## Data Availability

The data availability statement is not applicable to this study. The present study is a systematic review and data availability is not applicable.
